# Sudden hearing loss due to internal auditory canal metastasis of Her2-positive gastric cancer: A case report

**DOI:** 10.3892/ol.2014.2058

**Published:** 2014-04-10

**Authors:** CHANG-HEE KIM, JUNG EUN SHIN, HONG GEE ROH, JONG SIK LEE, SO YOUNG YOON

**Affiliations:** 1 Department of Otorhinolaryngology-Head and Neck Surgery, Konkuk University Medical Center, Konkuk University School of Medicine, Seoul 143-729, Republic of Korea; 2 Department of Radiology, Konkuk University Medical Center, Konkuk University School of Medicine, Seoul 143-729, Republic of Korea; 3 Division of Hematology-Oncology, Department of Internal Medicine, Konkuk University Medical Center, Konkuk University School of Medicine, Seoul 143-729, Republic of Korea

**Keywords:** stomach neoplasm, Her2/neu receptor, leptomeningeal carcinomatosis, hearing loss

## Abstract

Internal auditory canal (IAC) metastasis due to leptomeningeal carcinomatosis (LMC) from gastric cancer (GC) has rarely been reported. Early manifestation of symptoms, such as hearing loss, vertigo and facial paralysis, in cases of IAC metastasis due to LMC may facilitate the early detection of brain metastasis. To the best of our knowledge, the present study is the first to report IAC metastasis due to LMC in human epidermal growth factor receptor 2 (Her2)-positive GC. This study reports a case of an Her2-positive GC patient with LMC including IAC metastasis, who presented with acute sensorineural hearing loss, ipsilateral facial paralysis and vertigo during trastuzumab containing chemotherapy. The current study also discusses the early diagnosis and management of this complicated condition, demonstrating that clinical suspicion is key for a prompt diagnosis and proper management of LMC including IAC metastasis in Her2-positive GC.

## Introduction

Gastric cancer (GC) is the most common malignancy and the third most common cause of cancer-related mortality in Korea (1). Although leptomeningeal carcinomatosis (LMC) does not occur as frequently as with other malignancies, such as lung and breast carcinoma, gastric cancer may follow a complex course including metastasis to the brain parenchyma or meninges (2). Irrespective of the primary site of the cancer, LMC is a rare but devastating complication. The prevalence of LMC in GC is as low as 0.1–0.24% (3,4). Internal auditory canal (IAC) metastasis from GC has rarely been reported (5) and, to the best of our knowledge, IAC metastasis due to LMC has never been reported in GC.

The rate of Her2 positivity in GC is 22%, which is similar to the rate of that in breast cancer (BC) (6). Although increasing incidences of central nervous system (CNS) metastases have been reported in Her2-overexpressing BC due to the high anticancer effect of systemic trastuzumab (Her2 monoclonal antibody) without penetration of the blood-brain barrier, it is uncertain whether Her2 positivity in GC increases the risk of CNS metastases (7). LMC in Her2-positive GC has rarely been reported (8). The patient in the present case experienced LMC including IAC metastasis from Her2-positive GC. To the best of our knowledge, this is the first report of IAC metastasis in Her2-positive GC. Consent was obtained from the family of the patient.

## Case report

A 56-year-old male who had been diagnosed with Her2-positive advanced GC with multiple lymph node metastases, including left supraclavicular, paraaortic and retroperitoneal lymph nodes, underwent 12 cycles of combination chemotherapy composed of trastuzumab (Her2 monoclonal antibody), capecitabine and cisplatin at the Konkuk University Medical Center (Seoul, Korea). Cisplatin was discontinued after the eighth cycle due to cumulative peripheral neuropathy. Only capecitabine and trastuzumab were continued due to their clinical benefits. The response was a partial remission according to Response Evaluation Criteria In Solid Tumors (9).

**Figure 1:**
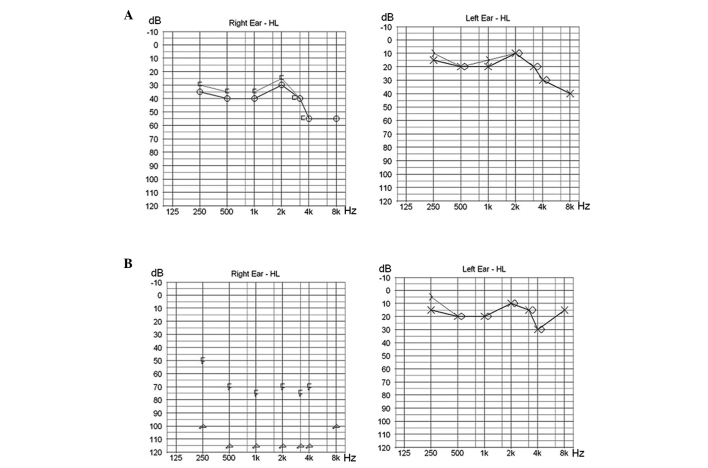
Pure tone audiometry after the 10th cycle of chemotherapy revealed (A) mild sensorineural hearing loss and (B) total sensorineural hearing loss on the right side at 2 weeks following the completion of 12 cycles of chemotherapy. HL, hearing loss.

After the 10th cycle of chemotherapy, the patient was referred to the Department of Otorhinolaryngology-Head and Neck Surgery at the Konkuk University Medical Center, Konkuk University School of Medicine (Seoul, Korea) for an evaluation of right ear fullness, which had been persisting for 15 days. Otitis media with effusion was diagnosed by an otoendoscopic examination, and the nasopharyngeal examination revealed no specific finding. Bone conduction pure tone audiometry after the treatment of the otitis media with effusion revealed mild (35dB) sensorineural hearing loss on the right side (Fig. 1A).

**Figure 2:**
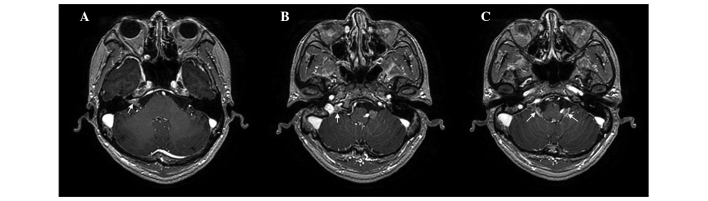
Magnetic resonance imaging of the brain showed: (A) Irregular nodular enhancement within the right internal auditory canal (arrow); (B) an enhancing lesion in the right jugular fossa (arrow); and (C) an enhancing mass in the bilateral cerebellomedullary cisterns (arrows).

Two weeks later, the patient suddenly developed a total hearing loss (Fig. 1B) and facial weakness on the right side, accompanied by severe vertigo. Physical examination revealed left-beating spontaneous nystagmus and peripheral-type facial palsy of House-Brackmann grade IV on the right side (10). Magnetic resonance imaging (MRI) of the brain revealed enhancing lesions in the right IAC (Fig. 2A), right jugular fossa (Fig. 2B) and bilateral cerebellomedullary cistern (Fig. 2C), and multifocal small enhancing nodules in other brain regions. The patient was diagnosed with LMC, and received whole-brain radiotherapy and palliative chemotherapy containing irinotecan, which is known to penetrate the blood-brain barrier (11). Following whole-brain radiotherapy, neurological symptoms including facial palsy and vertigo andnystagmus were improved, although the right hearing loss did not recover. However, the patient succumbed to rapid aggravation of his systemic metastases 8 weeks following the diagnosis of LMC with IAC. The interval from the date of diagnosis of GC to the date of diagnosis of LMC was 7 months. The patient’s survival time following the diagnosis of advanced GC was 9 months.

## Discussion

Sudden hearing loss, vertigo and ipsilateral facial palsy caused by IAC metastasis are uncommon initial presenting symptoms in patients with LMC. Recently, IAC metastasis due to direct invasion into the IAC from the metastatic lesion in the petrous apex was reported, which was a different spread pattern to that in the present case (5). LMC, hematogenous dissemination, and direct extension from the adjacent areas are considered as the possible routes of IAC metastasis (12). The present study reports the case of a patient with Her2-overexpressing GC who developed IAC metastasis and LMC despite systemic partial remission status with trastuzumab containing chemotherapy. This clinical condition may be the result of the neurotropism of Her2-overexpressing GC cells, as trastuzumab cannot penetrate th blood-brain barrier (13,14).

Early diagnosis of LMC is important in order to prevent irreversible neurological deficits. The most common symptoms of IAC metastasis include facial nerve palsy, hearing loss and tinnitus, and these symptoms are rapidly progressive in nature (12). Although a diagnosis of LMC including IAC involvement was not difficult to determine in this patient, the diagnosis would have been made earlier if the brain MRI had been performed when the patient showed mild sensorineural hearing loss. Sensorineural hearing loss, although mild, on the right side may have been caused by metastatic invasion into the IAC in this patient. In the present study, LMC was not suspected earlier as the patient developed LMC despite primary tumor and lymph node metastases which were controlled well by chemotherapy. The mild hearing impairment was not considered to be associated with the LMC, but instead due to cisplatin-induced ototoxicity or isolated ear problems. This case showed that the systemic response and response of the brain or leptomeninges may differ. Systemically well-controlled Her2-positive GC may develop as LMC and therefore, mild hearing impairments must be considered as a possible sign of LMC or brain metastases as prompt suspicion and timely therapy may improve patient outcome. This finding suggests that physicians should be attentive to patients with stable systemic disease for the timely diagnosis of LMC, rather than solely focusing on patients with systemically progressive disease or terminal disease status.

LMC in Her2-positive GC was first reported by Cavanna *et al* (8). Despite whole-brain radiotherapy and intrathecal (IT)trastuzumab, the patient died 3 months after the diagnosis of LMC. The patient in the present case also died within months (2 months) of the diagnosis of LMC. In the study by Cavanna *et al*, the researchers reported LMC and confusion, cognitive impairment and ataxia without hearing loss, which are different symptoms to those identified in the present case. IT trastuzumab therapy has been investigated in Her2-positive BC, and case reports indicate that this is a promising experimental treatment for patients with symptomatic LMC (7,15,16). However, it is unclear whether IT trastuzumab may have a clinical benefit in LMC of Her2-positive GC. In the present case, IT trastuzumab was not administered due to the patient’s general condition; rapid aggravation of the patient’s systemic metastases occurred immediately following discontinuation of chemotherapy. Aggressive treatment of LMC is particularly justified in patients whose disease is systemically well-controlled. Lapatinib, a tyrosine kinase inhibitor that targets Her2, has demonstrated clinical activity against CNS metastases in Her2-overexpressing BC (17). Lapatinib may be a therapeutic option for LMC in Her2-positive GC. Early diagnosis and optimal treatment of LMC including IAC metastasis is a crucial challenge. Future studies are warranted to establish optimal treatment of LMC in Her2-positive GC.

In conclusion, a clinical history of sudden sensorineural hearing loss raises the possibility of IAC metastasis, which may be a consequence of LMC, in Her2-positive GC. Prompt suspicion and an MRI are greatly helpful in the diagnosis of metastatic carcinoma to the IAC. We would like to emphasize that if hearing impairment is suspected in Her2-positive GC, LMC should be considered as a differential diagnosis, and early diagnosis and concerted therapeutic efforts are important for the management of LMC in Her2-positive GC.
